# Normal Human Pregnancy Results in Maternal Immune Activation in the Periphery and at the Uteroplacental Interface

**DOI:** 10.1371/journal.pone.0096723

**Published:** 2014-05-20

**Authors:** Andrea I. Loewendorf, Tina A. Nguyen, Maria N. Yesayan, Daniel A. Kahn

**Affiliations:** Division of Maternal-Fetal Medicine, Department of Obstetrics and Gynecology, David Geffen School of Medicine, University of California Los Angeles, Los Angeles, California, United States of America; New York University, United States of America

## Abstract

Pregnancy poses a unique challenge to the human immune system: the semi-allogeneic fetus must be protected from maternal immune attack while immunity towards pathogens is maintained. Breakdown in maternal-fetal tolerance can lead to pregnancy-specific diseases with potentially high degrees of morbidity and mortality for both the mother and her fetus. Various immune cell-types could mediate these functions, but a comprehensive evaluation of the peripheral and local maternal T cell and regulatory T cell compartments in normal human pregnancy is lacking. In this case-control study, we apply the Human Immunology Project Consortium proposed gating strategies to samples from healthy 3^rd^ trimester human subjects compared with healthy non-pregnant controls. The proportions of HLA-DR+ and CD38+ effector- and effector memory CD8 T cells are significantly increased in the peripheral blood of pregnant women. Utilizing a novel technique that takes advantage of the standard protocol for intrauterine cleanup after cesarean section, we isolate lymphocytes resident at the uteroplacental interface (UPI). At the UPI, the CD4 and CD8 T cell compartments largely mirror the peripheral blood, except that the proportion of HLA-DR+ activated T regulatory cells is significantly increased in direct proportion to an observed increase in the number of activated CD8 T cells. We find that cryopreservation and delayed sample processing (>12 hours) decreases our ability to identify regulatory T cell subsets. Further, the Consortium proposed method for Treg identification underrepresents Resting and Cytokine Tregs compared with Activated Tregs, thus skewing the entire population. Better understanding of the changes in the immune system during pregnancy in the peripheral blood and at the uteroplacental interface are essential for progress in treatment of pregnancy diseases such as pre-eclampsia and recurrent miscarriage.

## Introduction

Among the many challenges that face human immunology, the interplay between the immune systems of the pregnant mother and her semi-allogeneic fetus is extraordinarily complex, but of critical importance. Interaction takes place at the site of placental attachment to the uterus where the maternal decidua develops. Maternal adaptive immune cells are known to reside in the decidua; hence, local immune regulation must prevent maternal attack of her fetus. Multiple research groups have identified Regulatory T cells (Tregs) in early pregnancy decidua [1]–[4].

The study of the human immune system is subject to variability not seen in model organisms. To compensate for these anticipated irregularities, the Human Immunology Project Consortium proposed standardized antigen combinations to facilitate the identification of lymphocyte subsets and proposed recommendations for sample handling [5]. In this study, we apply those techniques to understand changes to the maternal immune system as a result of pregnancy. First proposed by Lanzavecchia, *et al.*, surface expression of the chemokine receptor CCR7 and the CD45-splice form CD45RA allows for the identification of four major T cell subtypes with different activation history and effector function biology [5], [6]. Additional use of CD45RO assists in the delineation of naïve from memory T cells [7] (**Table 1**).

**Table 1:** Human Immunology Project Consortium T cell subset identification markers.

	CCR7	CD45RA	CD45RO
Effector	negative	**positive**	negative
Naïve	**positive**	**positive**	negative
Effector memory	negative	negative	**positive**
Central memory	**positive**	negative	**positive**

The staining panels proposed by the Consortium are designed for application in a wide range of human conditions and thus also incorporate recommendations for surface proteins that would allow identification of activated T cells within the four different major subtypes. While the selection of “a single activation marker” that universally applies to both activated CD4 and CD8 T cells in different types of environments may not be possible, the Consortium suggests the use of HLA-DR and CD38 [8]–[10].

Tregs are the subject of ongoing intense research given their involvement as master regulators of immune homeostasis [10], [11]. The most definitive measure of Treg identification is via intra-nuclear FoxP3-staining. This poses a technical challenge, as the detection of FoxP3 requires lymphocyte fixation and permeabilization [12]. Such a process renders the cells not only dead, but also unusable for many downstream applications such as RNA isolation. Therefore, comprehensive identification of Tregs through a cell-surface marker approach is of considerable interest in human immunology. The Consortium proposes a combination of CCR4+CD25^hi^CD127^lo^ for the identification of Tregs [5].

In this case-control study, we apply the Consortium-proposed antigen selection for the identification of main T cell subsets (including Tregs) and their activation statuses to a comprehensive evaluation of the peripheral blood of healthy 3^rd^ trimester pregnant women compared to healthy non-pregnant women. In a second set of subjects, peripheral blood and maternal lymphocytes taken from the uteroplacental interface of healthy 3^rd^ trimester pregnant women were evaluated with the same methods. Since facilitation of the development of standardized flow cytometric assays is among the central goals of our report, we deliberately display the gating strategies for the breakdown of major T cell subsets and Tregs. Lastly, since human biological samples are often difficult to collect, particularly when connected with pregnancies, we report on the effects of delayed sample analysis and cryopreservation on the subsequent standardized assays.

## Materials and Methods

### Ethics Statement

Human subjects were recruited for participation under an IRB approved protocol (University of California, Los Angeles, Office of Human Research Protection Program, Medical IRB Committee-1 #11-003962). Each subject provided written informed consent prior to enrollment.

### Cryopreservation of Cells

Cells were enumerated, re-suspended at 5×10^6^ cells in 500 µl of 90% FBS (Gemini Bio, Sacramento, CA) and 10% DMSO (Fisher Scientific, Pittsburgh, PA) and placed into Cryo Nalgene Freezing Containers at –80°C overnight, then transferred to liquid nitrogen the next day.

### Human Subjects

Healthy non-pregnant and healthy women with uncomplicated, 3^rd^ trimester, singleton pregnancies were recruited for participation between April 2013 and March 2014. Demographic and obstetrical characteristics from the recruited population are provided in **Tables 2** and **3**.

**Table 2:** Human subject demographics**.**

Maternal (N = 18)		Non-pregnant Controls (N = 10)		P value
Age		Age		
Range	20–42	Range	21−37	p = 0.088 CI (−8.725−0.609)
Average	32.3	Average	28.5	
Median	32	Median	28.5	
Ethnicity		Ethnicity		
Caucasian	50%	Caucasian	60%	
Hispanic	39%	Hispanic	10%	
Black	6%	Black	0%	
Asian	6%	Asian	30%	
Gravity	2.6	Gravity	0.2	
Parity	0.9	Parity	0.2	
Smoker	20%	Smoker	0%	
Comorbidities		Comorbidities		
GDMA	22%		0%	

**Table 3:** Obstetric characteristics.

Unlabored	72%
Labored	28%
Vaginal Delivery	22%
FAD/VAD	0%
VBAC	6%
Primary Cesarean	28%
Repeat Cesarean	44%
Fetal Gender	
Male	Male 44%
Female	Female 56%
Average weight (g)	3538.94 g
APGAR-1 minute	8.33
APGAR-5 minute	8.94

### Tissue Collection

Peripheral blood (5–20 ml) was collected into EDTA-containing tubes using standard aseptic venipuncture technique, usually in conjunction with placement of a standard IV line. Cord blood was collected by after delivery of the fetus, but prior to placental separation into sterile cord blood collection kit containing Citrate Phosphate Dextrose solution (Medsep Corporation, Covina, CA). Cells from the uteroplacental interface were obtained as follows: At the time of cesarean section, after delivery of the fetus and placenta, the hysterotomy was wiped clean of blood. Then, a sterile surgical sponge was used to wipe the intrauterine cavity, a standard procedure that ensures complete removal of the placenta. This surgical sponge was placed into a container with ∼50 ml of sterile PBS. Although contamination of the sample with maternal peripheral blood or cord blood cannot be excluded, the phenotype of NK and T cells obtained with this sample collection method differed from both cord blood and maternal peripheral blood.

### Lymphocyte Purification

Granulocytes were depleted utilizing the RosetteSep Granulocyte Depletion Cocktail (Stemcell Technologies, Vancouver, Canada) following the manufacturer’s recommendations. Approximately 50 ml PBS wash of uteroplacental swipe were treated with 250 µl of Cocktail, granulocyte depletion of cord blood was performed with 1 ml of cocktail per 30 ml cord blood/Anticoagulant Citrate Phosphate Dextrose solution from the sterile cord blood collection unit. Granulocyte-depleted mononuclear cells were isolated by gradient centrifugation over Ficoll-Paque PLUS from GE Healthcare (Uppsala, Sweden) following the manufacturer’s recommendations. Cells were washed twice with sterile PBS and enumerated utilizing an Accuri flow cytometer with Propidium Iodide exclusion of dead cells.

### Phenotypic Analysis via Multicolor Flow Cytometry

Immediately after cell purification, 1×10^6^ cells were stained with Fixable Viability Dye eF780 (eBioscience) in 200 µl PBS in a 96 well u-bottom plate. The cells were pelleted and supernatant removed. Antibodies against surface antigens were added in 100 µl PBS and 1% FBS at the optimal concentrations determined by previous titration (**Table 4** **–** **6**) and incubated for 15 minutes at room temperature in the dark. Following two washes with PBS and 1% FBS, intra-nuclear FoxP3 staining was performed utilizing the FoxP3/Transcription Factor Staining Kit (eBioscience, San Diego, CA) per manufacturer’s instructions including the recommended 15 min incubation with mouse serum prior to intra-nuclear staining. Cells were re-suspended in PBS and 1% FBS, transferred to FACS tubes and analyzed within 6 hours. Either isotype staining or Fluorescence Minus One (FMO) stains were performed. For isotype control staining, the appropriate antibody isotype or, if unavailable, a similar isotype coupled to the same fluorophore was used at the same concentration as the antibody. Histogram gating was performed using the isotype staining as a guide to set the gates. For FMO controls, all antibodies minus one (FoxP3) were stained [13]. Analysis was performed on a BD SORP LSR II analytic flow cytometer with post-acquisition analysis performed with FlowJo (Treestar, Palo Alto, CA).

**Table 4:** Antibodies and flow cytometer settings used for antigen detection.

Antigen	Source	Clone	ng/test	Fluorophore	Excitation	Longpass Dichroic Mirror	Bandpass filter
CD45RO	Biolegend	UCHL1	200	Brilliant violet 421	405 nm	blank	450/50
CD4	Biolegend	OKT4	100	Brilliant violet 510	405 nm	505LP	525/50
CD25	Biolegend	BC96	250	Brilliant violet 605	405 nm	595LP	605/40
CD127	Biolegend	A019D5	300	Brilliant violet 650	405 nm	635LP	660/40
HLA-DR	Biolegend	L243	180	Brilliant violet 711	405 nm	685LP	710/50
CD196/CCR6	Biolegend	G034E3	500	Brilliant violet 785	405 nm	750LP	780/60
CD45RA	Biolegend	HI100	400	AF488	488 nm	505LP	530/30
CD3	eBioscience	UCHT1	300	PerCP-Cy5.5	488 nm	685LP	695/40
CD8	Biolegend	HIT8a	2500	AF700	640 nm	685LP	710/50
Viability	eBioscience		1∶1000	eF780	640 nm	755LP	780/60
CD38	Biolegend	HB7	150	APC	640 nm	blank	670/30
FoxP3	BD	259D/C7	500	PE	561 nm	blank	582/15
CD197/CCR7	BD	150503	500	PE-CF594	561 nm	600LP	610/20
CCR4	Biolegend	TG6/CCR4	300	PE/Cy7	561 nm	750LP	780/60

**Table 5:** Antibodies and flow cytometer settings used for antigen detection.

Antigen	Source	Clone	ng/test	Fluorophore	Excitation	Longpass Dichroic Mirror	Bandpass filter
CD56	Biolegend	HCD56	400	Brilliant violet 711	405 nm	685LP	710/50
Viability	eBioscience		1∶1000	eF780	640 nm	755LP	780/60
CD3	BD Horizon	UCHT1	5µl	PE-CF594	561 nm	600LP	610/20

**Table 6:** Antibodies and flow cytometer settings used for antigen detection.

Antigen	Source	Clone	ng/test	Fluorophore	Excitation	Longpass Dichroic Mirror	Bandpass filter
CD4	Biolegend	OKT4	100	Brilliant violet 510	405 nm	505LP	525/50
CD3	Biolegend	OKT3	250	Brilliant violet 605	405 nm	595LP	605/40
FoxP3	BD	259D/C7	500	AF488	488 nm	505LP	530/30
T-bet	Biolegend	4B10	100	PerCP-Cy5.5	488 nm	685LP	695/40
CD8	Biolegend	HIT8a	2500	AF700	640 nm	685LP	710/50
Viability	eBioscience		1∶1000	eF780	640 nm	755LP	780/60

### Statistical Analysis

Normality was determined using Shapiro-Wilk test. Differences in normally distributed populations were statistically analyzed using unpaired Student’s T test or analysis of variance (ANOVA) with Bonferroni/Dunn post-test. Analysis was accomplished with PRISM (Graphpad, La Jolla, CA).

## Results

### Pregnancy did not Alter the Proportion of Naïve-, Effector-, Effector Memory- or Central Memory T Cells in Peripheral Blood

**Figure 1:**
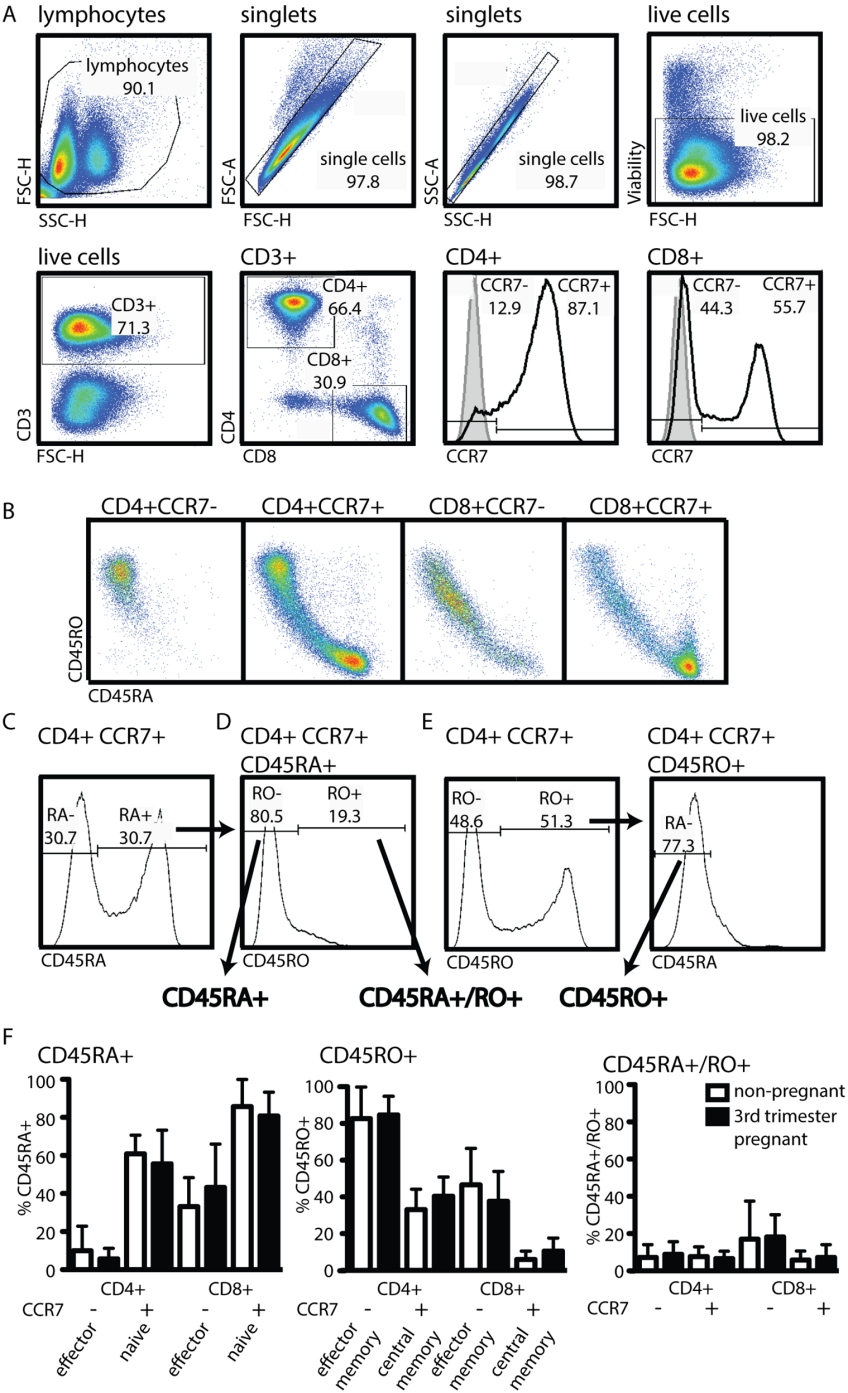
Pregnancy does not alter the proportions of effector, naïve, or memory T cells in the peripheral blood. Fresh peripheral blood from 9 healthy 3^rd^ trimester pregnant women and 9 healthy non-pregnant women was processed and analyzed as described in materials and methods. (A) Gating strategy for the identification of CD4+ and CD8+ T cells in maternal peripheral blood is depicted in respective dot blots. Positive populations of CCR7 are determined with the help of isotype staining (full grey histogram) or antibody staining (black line) on total CD4 or CD8 populations. (B) Dot blot depiction of CD45RA and RO in the four sub-populations identified in the two histograms in A. (C–E) Gating strategy for the identification of three T cell sub-populations with distinct history. (C) CD45RA expression defining CD45RA+ cells (gate set based on isotype control staining). (D) CD45RO expression is depicted in these CD45RA+ cells and the CD45RO− cells are identified (CD45RA+, CD45RO−) as the CD45RA+ population. The CD45RO+ cells of that same histogram are identified as CD45RA+/RO+. (E) CD45RO+ cells are defined as CD45RO+ in a histogram (*left*, gate set based on isotype control staining) and the cells expressing CD45RO are gated for the absence of CD45RA in another histogram gating (*right*). Statistical analysis was done using a non-paired two-tailed Student’s T test.

Pregnancy poses an exceptional challenge to the maternal immune system: the need for effective immunity to pathogens while maintaining active tolerance of her fetus. To understand the impact of this delicate immune balance on the maternal immune profile, we asked whether pregnancy induces skewing of the CD4 and CD8 T cell compartments toward a specific sub-type (e.g., effector T cells). We employed recommendations by the Consortium for the identification of lymphocyte subsets in adult peripheral blood [5]. Histogram analysis was used to determine CCR7 positive and negative populations (**Figure 1A**). Co-staining of T cells with CD45RA (naïve) and CD45RO (memory) revealed a non-exclusive expression pattern of these two isoforms (**Figure 1B**). To account for this fact, we deviated from the Consortium recommendations and employed a two-step histogram gating strategy that identifies CD45RA+ T cells (**Figure 1C**) but excludes CD45RO+ T cells from this population (**Figure 1D**, CD45RA+CD45RO−, henceforth CD45RA+). Cells expressing both CD45RA and CD45RO were identified in the same histogram (**Figure 1D**, CD45RA+/RO+). Similarly, to identify cells that exclusively express CD45RO, CD45RA+ cells were excluded (**Figure 1E**, CD45RO+CD45RA−, henceforth CD45RO+). Comparison of CCR7− and CD45RA− expression in both CD4 and CD8 T cell compartments between 3^rd^ trimester pregnant and non-pregnant women did not reveal a statistically significant difference **(** **Figure 1F** **)**.

### The Percentage of Tregs in the Peripheral Blood does not Change with Pregnancy

**Figure 2:**
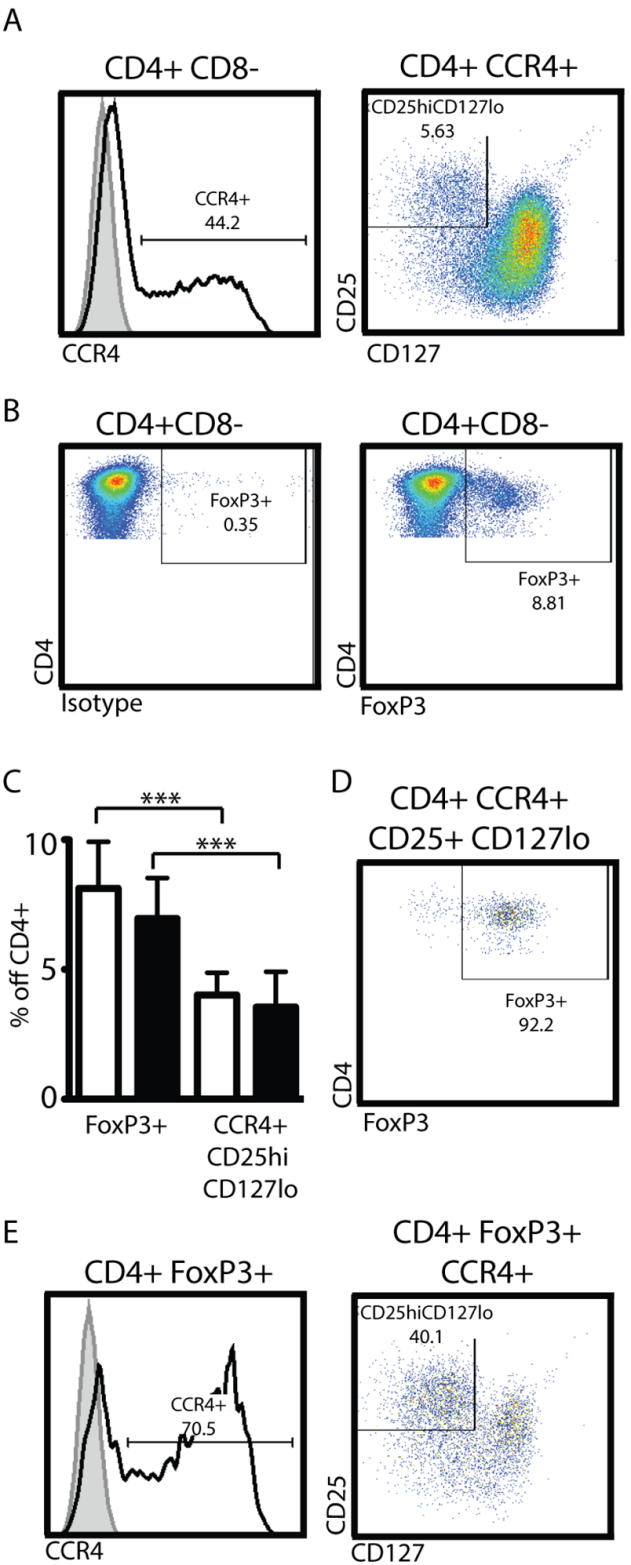
Less than 50% of FoxP3+ CD4+ T cells are CCR4+ CD25^hi^ CD127^lo^. Fresh peripheral blood from healthy 3^rd^ trimester pregnant women or healthy non-pregnant women was processed and analyzed as described in materials and methods. CD4+CD8− cells were identified as depicted in Figure 1. Stained maternal cells were subjected to two different gating strategies to identify regulatory T cells. (A) CCR4+ CD4+ T cells were identified with the help of isotype staining (full grey histograms, total CD4 T cells) or antibody staining (black line). Depiction of CD4+CCR4+ in a CD25 vs. CD127 dot blot allows for the identification of CD25^hi^ CD127^lo^ cells (A, *right*). (B) Identification of FoxP3+ cells via intra-nuclear FoxP3-staining: left panel isotype FMO staining, right panel FoxP3 antibody staining. (C) Proportion of Treg identified via FoxP3+ or surface staining. White bars: peripheral blood of healthy non-pregnant women. Black bars: peripheral blood of healthy 3^rd^ trimester women. (D) CCR4+CD25^hi^CD127^lo^ cells identified in A were subjected to FoxP3+ gating as in B. (E) FoxP3+ cells identified in B were subjected to gating for CCR4+CD25^hi^CD127^lo^ cells as in A. Statistical analysis was done using a non-paired two-tailed Student’s T test.

The Consortium proposed a combination of high-expression of CD25 and low expression of CD127 (CD4+CCR4+CD25^hi^CD127^lo^) for the identification of Tregs. We applied this proposed staining and gating strategy to peripheral blood from non-pregnant and healthy 3^rd^ trimester pregnant women (**Figure 2**). We also included an antibody against the intra-nuclear FoxP3 protein in our staining panel, allowing for the identification of Tregs via both marker combinations in the same sample. Display of CD4+CCR4+ cells in a CD25 vs. CD127 dot blot reveals a distinct population of CD25^hi^CD127^lo^ cells that can be clearly identified for accurate gate placement (**Figure 2A**, *right*). To identify FoxP3+ T cells, Fluorescence Minus One (FMO)-isotype staining is used for gate placement (**Figure 2B** **)**. Applying these two gating strategies to the same set of subject samples, we did not find a difference in the percentage of Tregs found in the peripheral blood of non-pregnant vs. 3^rd^ trimester pregnant women. However, the percentage of Tregs identified via surface staining (CD4+CCR4+CD25^hi^CD127^lo^) was significantly lower than the percentage identified via FoxP3 intra-nuclear antibody staining (**Figure 2C**). To characterize potential overlap between the two methods, we applied the FoxP3-gating strategy to the population identified via cell surface antigens and found that >85% of those cells were FoxP3+ (**Figure 2D** non-pregnant average = 87.35±1.84%, 3^rd^ trimester healthy pregnant average = 86.95±2.65%). When we applied the CD4+CCR4+CD25^hi^CD127^lo^ gating strategy to FoxP3+ cells, less-than 50% of the FoxP3+ cells fell within those gates (**Figure 2E** non-pregnant average = 43.37±4.04%, 3^rd^ trimester healthy pregnant average = 42.51±7.61%). Taken together, these observations indicated that cells identified via the CCR4+CD25^hi^CD127^lo^ gating strategy were largely FoxP3+, but the surface marker approach only identified a *minority* of the FoxP3+ population.

### Previously Frozen or >12 Hour Old PBMC are not Suitable for Intra-nuclear Foxp3-staining

**Figure 3:**
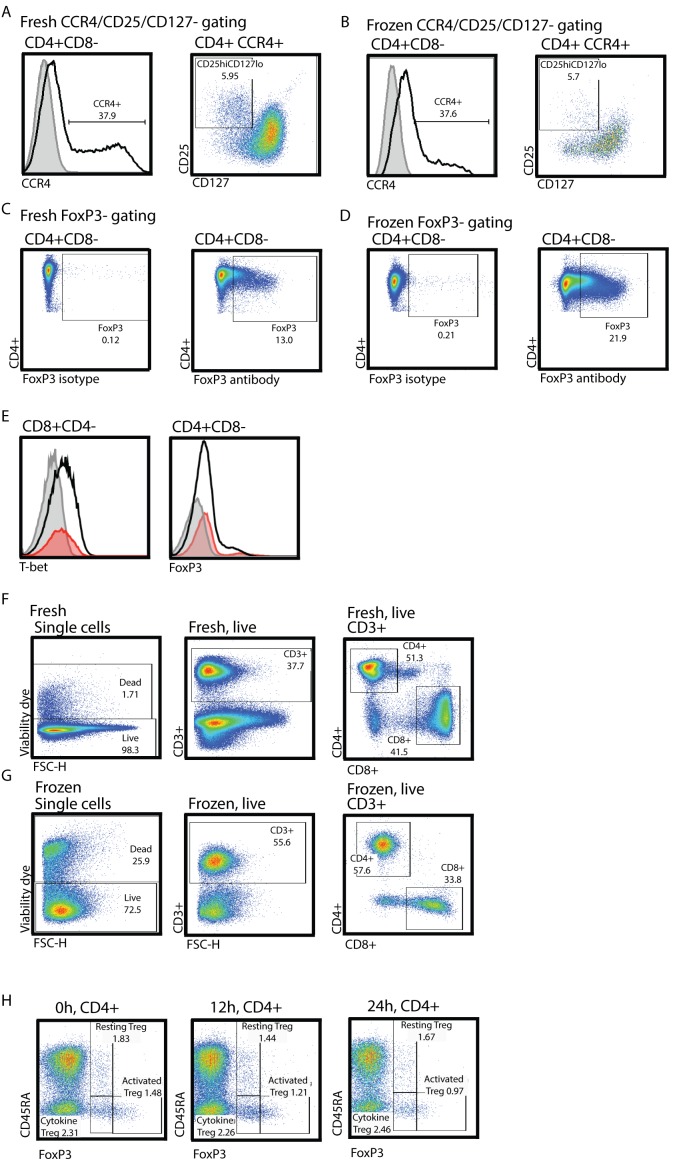
FoxP3-staining of frozen PBLs does not yield reliable results. Fresh (A, C, E, F) or previously frozen (B, D, E, G) peripheral blood lymphocytes from healthy 3^rd^ trimester pregnant women was processed and analyzed as described in materials and methods. CD4+CD8− cells were identified as depicted in Figure 1. (A and B) CD4+CD8− cells were gated for Treg identification via CCR4+CD25^hi^CD127^lo^ as in Figure 3A. (C and D) Identification of FoxP3+ cells via intra-nuclear FoxP3-staining. (E) Peripheral blood from non-pregnant donors fresh (red histogram) or previously frozen (grey histogram isotype staining, black histogram antibody staining) was stained with T-bet (left histogram) or FoxP3-antibody (right histogram). (F, G) Gating steps of fresh (F) or frozen (G) PBL. (I) Fresh (left dot blot), 12 h old (middle dot blot) and 24 h old (right dot blot) was processed and analyzed as described in materials and methods. Gates set based on FMO control.

Human samples obtained from rare conditions are often subjected to cryopreservation and/or delayed analysis given clinical constraints. To test the impact of common sample handling techniques, we performed Treg identification analysis (FoxP3-staining and CCR4+CD25^hi^CD127^lo^) on samples from the same subjects fresh, >12 hours, and after standard cryopreservation (**Figure 3**). Surface marker identification of Tregs (CCR4+CD25^hi^CD127^lo^) was comparable between fresh and frozen samples (**Figure 3A,B** respectively). In contrast, intra-nuclear FoxP3 staining of frozen samples from the same subject did not yield interpretable results. Cryopresevation results in 25% cell death with a disproportionate survival of CD3+ cells (**Figure 3F, G**). Among T cells, CD4 and CD8 proportions were not affected by cryopreservation (**Figure 3F, G**). Evaluation of intranuclear staining found that while the isotype staining was satisfactory, the FoxP3-antibody or T-bet staining was grossly over-stained (**Figure 3E**). We also tested the detection of FoxP3 by intra-nuclear staining in blood samples 24 hours and 12 hours post-collection (**Figure 3I** *middle and right*) in comparison to fresh blood samples (**Figure 3I** *left*). While the viability of older samples was comparable as assessed via fixable viability dye included in the staining panel, FoxP3-staining intensity was greatly diminished in older samples without clear separation between FoxP3+ and FoxP3- populations. Clear gating and identification of FoxP3+ cells was not possible due to this low staining intensity. The population predominately affected by delayed processing were the activated Treg populations.

### Pregnancy Induces CD38 and HLA-DR-expression in CCR7−CD8 T Cells

**Figure 4:**
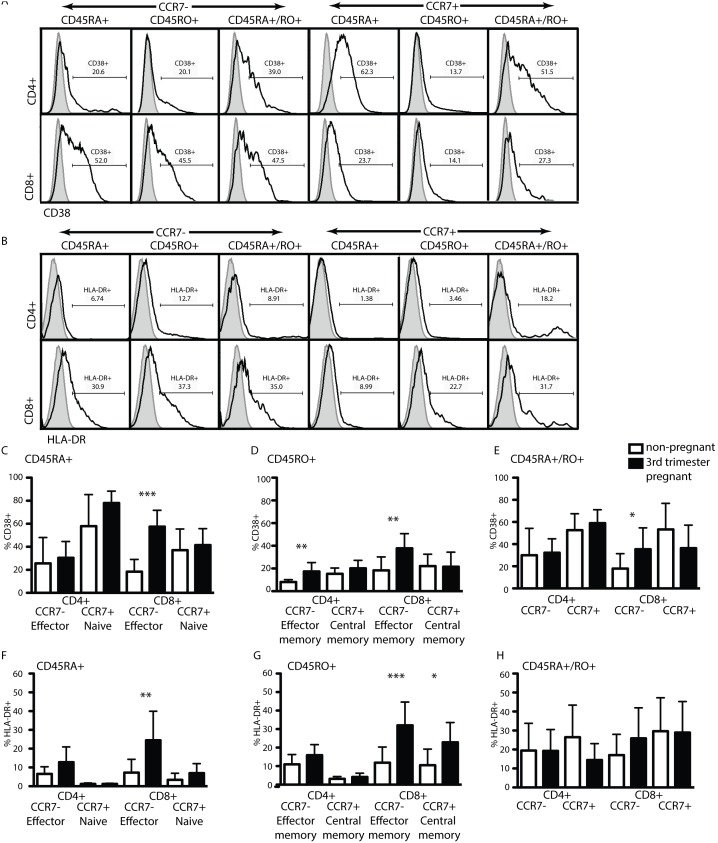
CD8+ T cells in the peripheral blood of healthy 3^rd^ trimester pregnant women are activated. Fresh peripheral blood from healthy 3^rd^ trimester pregnant women or non-pregnant women was processed and analyzed as described in materials and methods. CD4/CD8, CCR7+ or CCR7−, CD45RA+, CD45RO+ or CD45RA+/RO+ sub-populations were identified as depicted in Figure 1 and 2. (A) CD38 or (B) HLA-DR-expression of the different CD4 or CD8 sub-populations indicated above the histograms in maternal cells is determined with the help of isotype staining (full grey histograms, total CD4 or CD8 T cells) or antibody staining (black line, respective sub-population). (C–E) % of CD38+ or (F–H) HLA-DR+ cells falling into the CD45RA+ (C and F) CD45RO+ (D and G) or CD45RA+/RO+ (E and H) sub-populations off the respective parent gate as described in Figure 1. Statistical analysis was done using a non-paired two-tailed Student’s T test (*,**,***p<0.05).

As shown in **Figure 1** we did not find alterations in the T cell subtype repertoires as identified by CCR7 and CD45RA/RO discrimination, as a function of pregnancy. We next hypothesized that pregnancy induces a change in activation status of T cell subtypes. We probed for activation using antibodies against the surface proteins CD38 and HLA-DR in our staining panel (**Figure 4A, B**). We found no significant differences in the percentage of these populations between healthy non-pregnant women and 3^rd^ trimester pregnant women in all CD4 sub-populations analyzed, except for an enrichment of activated CD38+ CD4 effector memory cells (**Figure 4C–H**, *left*). However, both CD38 and HLA-DR were expressed by a significantly higher proportion of CD8+ effector- and effector memory T cells (**Figure 4F, G**) indicating activation of tissue-resident CD8 T cells independent of their history (naïve or memory).

### CD8+ T Cells are Significantly Enriched at the Uteroplacental Interface

**Figure 5:**
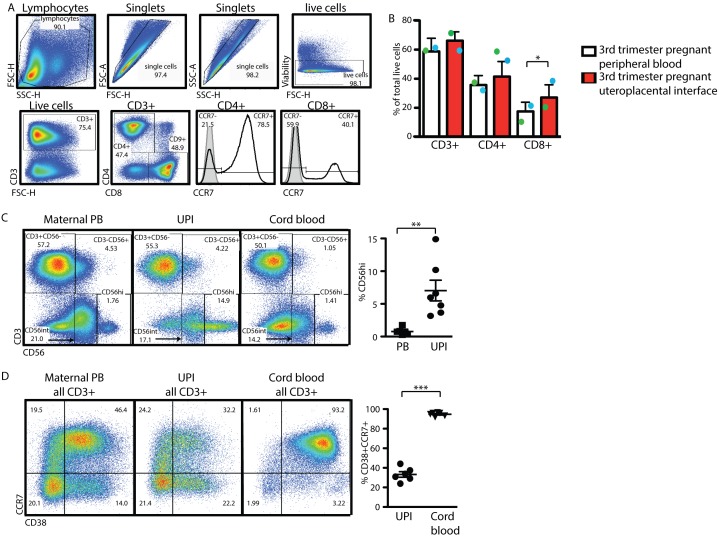
The uteroplacental interface displays a unique composition of lymphocytes. Positive populations of CCR7 are determined with the help of isotype staining (full grey histogram) or antibody staining (black line) on total CD4 or CD8 populations. (A) Lymphocytes of the UPI were analyzed in parallel with peripheral blood of the same subject, gates were set based on the populations in peripheral blood of the same subject. (B) Percentage of the live cells identified as CD3+, CD3+CD4+ or CD3+CD8+ in peripheral blood and UPI. (C) Identification of lymphocyte sub-populations in total live cells in maternal PB (left dot blot), UPI (middle dot blot) and matched cord blood (right dot blot). Percentage of CD3–CD56^hi^ cells in maternal PB and UPI (graph right hand) off total live lymphocytes. (D) Identification of CCR7+CD38+ T lymphocytes in total CD3+ cells in maternal PB (left dot blot), UPI (middle dot blot) and matched cord blood (right dot blot). Percentage of CCR7+CD38+ cells in maternal UPI and cord blood (graph right hand) off total CD3+ lymphocytes. Statistical analysis was done using a non-paired two-tailed Student’s T test (*,**,***p<0.05).

To comprehensively evaluate T cell changes during pregnancy, we sought to characterize T cell subsets at the UPI. The uterine site of placental attachment, the decidua, is the major site of physical interaction between the mother and fetus. To characterize the lymphocytes at this site, we utilized a novel method of human decidual lymphocyte sample collection. We were able to obtain between 1–3×10^7^ lymphocytes per subject from the decidua. We compared the composition of the lymphocyte microenvironment at the UPI to the peripheral blood within the same subjects (**Figure 5**). To ensure consistency, the same gating strategy and specific gates were applied to the peripheral blood and UPI lymphocytes (**Figure 5A**). Viability of the UPI lymphocyte preparations was consistently >90% (*data not shown*). We found CD8+ T cells to be significantly enriched at the UPI compared to the subject’s peripheral blood (mean CD8+ PB 17.52±6.374%. UPI mean 27.13±8.702%, *p = 0.0363* n = 10). To gauge potential contamination of the UPI sample with maternal peripheral blood and/or cord blood, we performed a co-staining of CD3 and CD56 to identify the major T-and NK cell subsets in maternal PB, UPI and matched cord blood (**Figure 5C**). We identified a significantly higher proportion of CD56^hi^CD3− cells in the UPI compared to the maternal PB (**Figure 5C**, *far right*). Staining of total CD3+ populations with CCR7 and CD38 allowed us to identify a CD38+CCR7+ population that comprises >90% of total cord blood T cells, a significantly higher proportion than what we find in the UPI (**Figure 5D**, *far right).* Taken together, we propose that contamination of UPI sample harvested as described by either cord blood or maternal PB is minimal.

**Figure 6:**
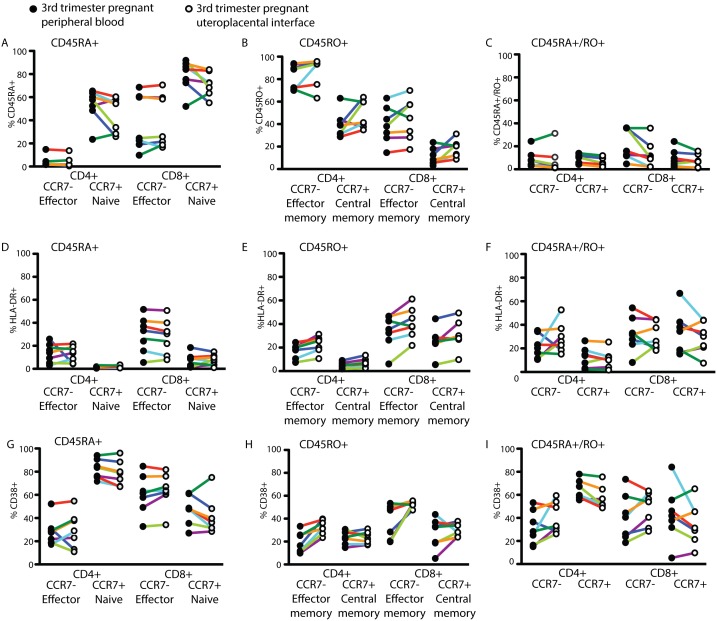
While CD8+ T cells are enriched at the uteroplacental interface, subset composition and activation status mirrors the peripheral blood. Percentage of the live cells identified as (A) CD45RA+, (B) CD45RO+, (C) CD45RA+/RO+, (D–F) HLA-DR+, or (G–I) CD38+ in the peripheral blood n = 7 (closed circles) and uteroplacental interface n = 7 (open circles) of the same subjects. Color coding of the subjects is consistent within and between Figures 6 and 7.

Utilizing the same gating strategy as described for **Figure 1**, we delineated the proportions of CD45RA, CD45RO and CD45RA+/RO+ cells within the CD4+ and CD8+ CCR7+ and CCR7− sub-populations in the peripheral blood and UPI of the same subjects (**Figure 6A–C** **).** Similarly, we assessed the activation status of the different T cell subsets by analyzing the proportion of HLA-DR+ (**Figure 6D–F**) and CD38+ (**Figure 6G–I**) cells. We did not find significant differences, suggesting that while the UPI is enriched in CD8+ T cells, the T cell subset composition and activation status of the UPI mirrors the peripheral blood.

### HLA-DR+ Treg Accumulate at the Uteroplacental Interface

**Figure 7:**
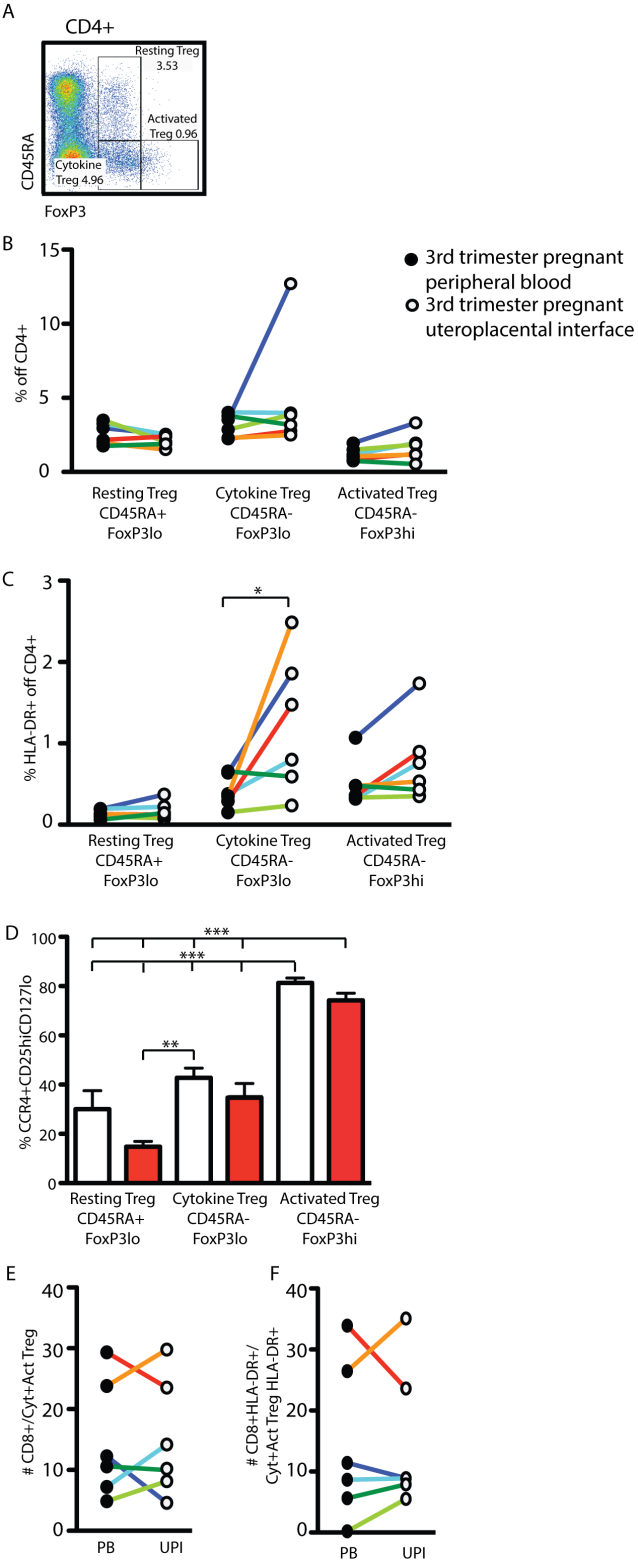
HLA-DR+ Cytokine Tregs accumulate at the uteroplacental interface. Fresh peripheral blood from healthy 3^rd^ trimester pregnant women and the uteroplacental interface was processed and analyzed as described in materials and methods. Gating strategy for the identification of CD4+CD8− T cells as depicted in **Figure 1A**. (A) Gating strategy for the identification of Resting Treg (CD45RA+FoxP3^lo^), Cytokine Treg (CD45RA−FoxP3^lo^) and Activated Treg (CD45RA−FoxP3^hi^) in the dot blot of CD4+CD8− cells. (B) Percentages of Resting, Cytokine, and Activated Treg within the CD4+CD8− T cells in the peripheral blood (closed circles) and uteroplacental interface (open circles) of the same subjects. (C) Percentage of HLA-DR+ Treg of the three indicated sub-groups within CD4+ T cells. (D) Percentage of FoxP3+ T cells of the indicated three sub-groups that fall within the CCR4+CD25^hi^CD127^lo^ gating strategy depicted in **Figure 3A**. (E) Number of CD8+ T cells per Cytokine-and Activated Treg in the peripheral blood (closed circles) and UPI (open circles). (F) Number of HLA-DR+CD8+ T cells per HLA-DR+ Cytokine-and Activated Treg in the peripheral blood (closed circles) and UPI (open circles). Statistical analysis was done using a non-paired two-tailed Student’s T test (B and C) and one-way ANOVA with Bonferroni-Dunn post-test (D) (*, ***p<0.05).

Sakaguchi *et al.*, [14] developed a simple staining and gating strategy that allows for the analysis of three Treg subtypes with distinct biological features (**Table 7**). Application of this gating strategy (**Figure 7A**) to lymphocyte specimens from the peripheral blood and UPI of healthy 3^rd^ trimester pregnant women illustrated no significant differences between the percentages of the three sub-populations (**Figure 7B**). HLA-DR-expression identifies a highly suppressive population [15], [16]; for that reason we analyzed the expression of this molecule on the surface of the three Treg subtypes in both subject groups. While only a few of the resting Tregs expressed this molecule, we found significant local enrichment of Cytokine Tregs at the UPI when compared with the peripheral blood (**Figure 7C**). While there was a similar trend in the composition of Activated Tregs, the increase was not significant.

**Table 7:** Treg subset identification strategy [14].

	FoxP3	CD45RA
Resting Treg	low	positive
Cytokine Treg	low	negative
Activated	high	negative

### Surface Staining Underestimates the Proportion of Resting Tregs

Surface staining (CCR4+CD25^hi^CD127^lo^) for the identification of Tregs significantly underestimates the number of FoxP3+ lymphocytes (**Figure 2C**). To address whether this underestimation is equally true for all Resting Treg, Cytokine Treg and Activated Treg, we applied the CCR4+CD25^hi^CD127^lo^ gating strategy to the three Treg sub-groups (**Figure 7D**). We found >70% of Activated Tregs were CCR4+CD25^hi^CD127^lo^. Surface identification of Resting Treg and Cytokine Tregs was significantly less than direct FoxP3-staining in both the peripheral blood and the UPI. Thus, within the Treg population identified by surface staining alone, Activated Treg are *over-*represented while Resting and Cytokine Tregs are specifically *under-*represented.

### The Ratio of CD8+ Responder T Cells to Treg is Consistent in the Peripheral Blood and the Uteroplacental Interface

Although the specific modes of action by which Tregs suppress immune responses are not yet clearly understood, the importance of a numeric relationship between responder cells and suppressor cells is apparent [17]. To address this ratio as a function of the microenvironment at the UPI vs. peripheral blood of the same subject, we calculated the number of CD8+ responder cells per suppressive Treg (Cytokine Treg + Activated Treg, **Figure 7E**). While we found considerable inter-subject variability in absolute numbers, the ratio of CD8+ T cells to Treg was remarkably similar in the peripheral blood and the UPI. Additionally, a comparison of the activated (HLA-DR+) sub-populations of both responders (HLA-DR+ CD8+) and suppressors (HLA-DR+ Cytokine Treg and Activated Treg) also demonstrated a remarkable consistency between the peripheral blood and the UPI (**Figure 7F**).

## Discussion

Mechanistic studies of immunologic phenomena in humans are challenging in part due to outbreeding. To facilitate reproducibility, the Human Immunology Project Consortium has proposed a framework for T cell subset identification [5]. We applied this framework to the unique situation of human pregnancy. To facilitate subsequent research, we deliberately present our primary data with details of the FACS gating strategies used.

We utilize a novel method to sample the UPI that should also be easily adopted by others. This simple, no-added risk, method recovers lymphocytes from the surgical sponge used to ensure complete placental removal after Cesarean section. Previous descriptions of human decidual lymphocytes has relied on either full thickness biopsies or samples from sharp decidual curettage [18]. Both of those procedures carry modest risk to the patient. The recovered lymphocyte composition and phenotype indicates that our sampling method does not include significant fetal-or maternal peripheral blood contamination.

We found that defining Treg as CCR4+CD25^hi^CD127^lo^ cells results in significant underestimation of lymphocytes that express FoxP3 in both pregnant and non-pregnant women. Second, our ability to appropriately characterize Treg subsets via intra-nuclear FoxP3 staining diminished once a sample had been left within the collection tube for more than 12 hours or had been cryopreserved. The latter was particularly concerning as the FMO control displayed an acceptable amount of background staining. We found that cryopreservation results in variable effects on intranuclear staining. That is, FoxP3 or T-bet stating results in antibody staining was much higher than in the previously performed staining using fresh cells of the same subject. This finding poses a special challenge for standardization as the most reliable staining control (FMO) fails to indicate this technical issue and therefore requires a well-trained eye to identify such a false-positive event. Hence, the reason we included the primary data for illustration. Our observations contrast to reports in the literature where different FoxP3-staining buffer kits and FoxP3-antibody clones, including the ones used in our study, have been tested on fresh and cryopreserved samples [19], [20]. The authors did not report over-staining in the cryopreserved samples but the gating strategy employed in these studies is based on a combination of FoxP3 and CD25, while our study focuses on FoxP3 to identify Treg subsets and hence is more dependent on clear population separation and accuracy of the FoxP3-antibody.

In contrast to the intra-nuclear FoxP3 staining, the combination of surface markers proposed by the Consortium was not negatively impacted by cryopreservation and thereby proves a more reliable method in circumstances when cryopreservation is essential. The clear downside of this method is the significantly lower proportion of FoxP3+ cells that are identified via surface definition. Further, not all sub-types of Tregs are affected equally. Resting and Cytokine Tregs are disproportionately *under*-identified versus Activated Tregs.

Transient expression of FoxP3 by activated T cells has been observed ([21]–[23]). We find an increase of T cells expressing activation markers such as HLA-DR and/or CD38 (**Figure 4** **)**. We cannot rule out that a proportion of the cells identified by FoxP3 are indeed effector cells with no suppressive capacity. Given the transient nature of FoxP3 expression in non-suppressive cells, we believe this proportion to be small but only suppression assays and FoxP3 promoter methylation assays can give insight into this.

Consequently, Treg populations identified via surface markers contain a much higher proportion of Activated Tregs than what the definitive FoxP3/CD45RA-staining would otherwise indicate.

We find variations in T cell subset compositions between subjects as would be expected in our small sample. While our sample size lacks the statistical power to define more subtle changes in T cell subsets, such differences may not have the same biological meaning as those significant differences we report here. That is, we find that the activation status of CD8+ T cells is significantly increased in 3^rd^ trimester pregnant women compared to non-pregnant women both in the periphery and at the local site of action (UPI). This increase is true for both CCR7− CD8+ T cell populations; the effector (CD45RA+) and effector memory (CD45RO+) T cells. Presumably, this increase in effector T cell activation is in response to the fetal allograft, but is held in check by maternal tolerance mechanisms, such as Activated and Cytokine Tregs. Interestingly, the increase in HLA-DR+ Cytokine Tregs at the UPI mirrored the increase in HLA-DR+ CD8 T cells resulting in a similar numeric relationship between responder CD8 T cells and suppressor cells at the UPI and in the peripheral blood. The degree to which this numerical relationship is a true indicator of the suppressive state of the UPI microenvironment is subject to future studies [2], [15], [24], [25]. Hypothetically, derangements in the ratio of HLA-DR+ Cytokine and Activated Tregs to HLA-DR+ CD8 T cells might be found in pathologic conditions such as recurrent pregnancy loss or pre-eclampsia.

We find that T cell subset composition and activation status at the UPI is comparable with that found in the peripheral blood. These observations are in contrast with other reports, which find an enrichment of effector memory CD8 T cells when sampling maternal decidua attached to the placenta rather than directly from the maternal structure [4]. Our observation of a significant increase of highly suppressive HLA-DR+ Treg in the decidua is consistent with previous reports from studies of the first trimester of pregnancy as wells as analyses of samples of decidua taken from the placenta [25]–[27].

## Conclusion

Since human pregnancy cannot be adequately studied in rodent models, careful mechanistic human studies are essential. The work we show here provides technical guidance towards better understanding T cell subset changes with pregnancy. These studies will be used to define further investigation into the immunology of pregnancy and its impact on diseases of pregnancy.
